# Influence of accuracy, repeatability and detection probability in the reliability of species-specific eDNA based approaches

**DOI:** 10.1038/s41598-018-37001-y

**Published:** 2019-01-24

**Authors:** Quentin Mauvisseau, Alfred Burian, Ceri Gibson, Rein Brys, Andrew Ramsey, Michael Sweet

**Affiliations:** 1 Aquatic Research Facility, Environmental Sustainability Research Centre, University of Derby, Derby, DE22 1GB UK; 2 Surescreen Scientifics Ltd, Morley Retreat, Church Lane, Morley, DE7 6DE UK; 3 Freshwater Biological Association, Ferry Landing, Far Sawrey, Ambleside, Cumbria LA22 0LP UK; 4 Research Institute for Nature and Forest, Gaverstraat 4, 9500 Geraardsbergen, Belgium

## Abstract

Environmental DNA (eDNA) barcoding has a high potential to increase the cost-efficiency of species detection and monitoring in aquatic habitats. However, despite vast developments in the field, many published assays often lack detailed validation and there is little to no commonly (agreed upon) standardization of protocols. In this study, we evaluated the reliability of eDNA detection and quantification using published primers and assays targeting the Freshwater Pearl Mussel as a model organism. We first assessed limits of detection for two different target genes (COI and 16S) following the MIQE guidelines, and then tested the reliability of quantification in a double-blind mesocosm experiment. Our results reveal that different methodological indicators, namely accuracy, repeatability and detection probability affected the reliability of eDNA measurement at the different levels tested. The selection of the optimal analytical method was mainly determined by detection probability. Both the COI and 16S assays were highly specific for the targeted organism and showed similar accuracy and repeatability, whilst the limit of detection was clearly lower for the COI based approach. In contrast, the reliability of eDNA quantification hinged on repeatability, reflected by the scattering (*r*^2^ = 0.87) around the relationship between eDNA and mussel density in mesocosms. A bootstrapping approach, which allowed for the assignment of measures associated with repeatability of samples, revealed that variability between natural replicates (i.e. accuracy) strongly influenced the number of replicates required for a reliable species detection and quantification in the field.

## Introduction

(Definition of terms related to eDNA assay validation; In our study, we refer to the following terms from an eDNA perspective):

Reliability: Degree to which the result of all aspects of assay evaluation can be precise and repeatable.

Specificity: Correct amplification of the targeted species and no positive results from closely related species.

Detection probability: Probability that the analysis of a technical or natural replicate that contains DNA of the target species results in a positive detection.

Sensitivity: Synonymous with the Limit of Detection (LOD), which is according to the MIQE guidelines^1^ defined as the last dilution step of the standard that results in detection of the targeted DNA with a threshold cycle below 45.

Efficiency: Degree to which the amplification of all DNA copies, in all PCR reactions, can be precise and repeatable.

Repeatability of quantification: In an eDNA context standard curves are based on mean of measurements. Hence, repeatability represents the spread (r2) of data around regression lines used to standardise quantification.

Accuracy of quantification: Variability of measurements contributing to a data point. Includes both, variability in natural and technical replicates. At low replicate number, low accuracy is likely to decrease repeatability).

Environmental DNA (eDNA) is a novel molecular technique, which can facilitate via the analysis of water samples (in this context), the detection and monitoring of organisms and communities in aquatic habitats that are difficult to monitor with more traditional methods^2–5^. The technique is based on the amplification of fragments of DNA originating from skin, hairs, mucus or gametes for example, all of which can be shed by both living and dead organisms alike^2,6–10^. Assays can be either non-targeted (i.e. a metabarcoding approach) or targeted at specific species^11^. Further, the application of advanced amplification methods such as quantitative Polymerase Chain Reaction (shortened to qPCR, also known as real-time PCR) or digital droplet PCR (ddPCR also known as digital PCR) allows the quantification of target DNA in natural habitats. Accordingly, correlations between species abundance and eDNA detection and quantification has recently been demonstrated for several species^8,12–18^. However, a common limitation of many eDNA based quantification approaches is that only a few cases report rigorous validation steps at a satisfactory level under controlled laboratory conditions^13,16^. In many examples, validation steps which have been implemented simply depend on correlative comparison with field surveys^14,17,18^. Field surveys, however, have been shown to be often highly variable and underrepresent true species abundance and diversity^14^. Meaning, it could therefore be argued that we have little information on the reliability of eDNA assays with regard to quantifiable data.

The reliability of eDNA based quantification does not only depend on the repeatability and accuracy of quantification, but also on sensitivity, which is linked to the detection probability of any given approach (see definitions used within this study). For instance, the efficiency and reliability of qPCR assays depend on whether they follow the Minimum Information for Publication of Quantitative Real-Time PCR Experiments Guidelines or MIQE for short^1^. In particular, validation of any novel assays should at the very least highlight the Limit of Detection (LOD) and the Limit of Quantification (LOQ).

**Figure 1:**
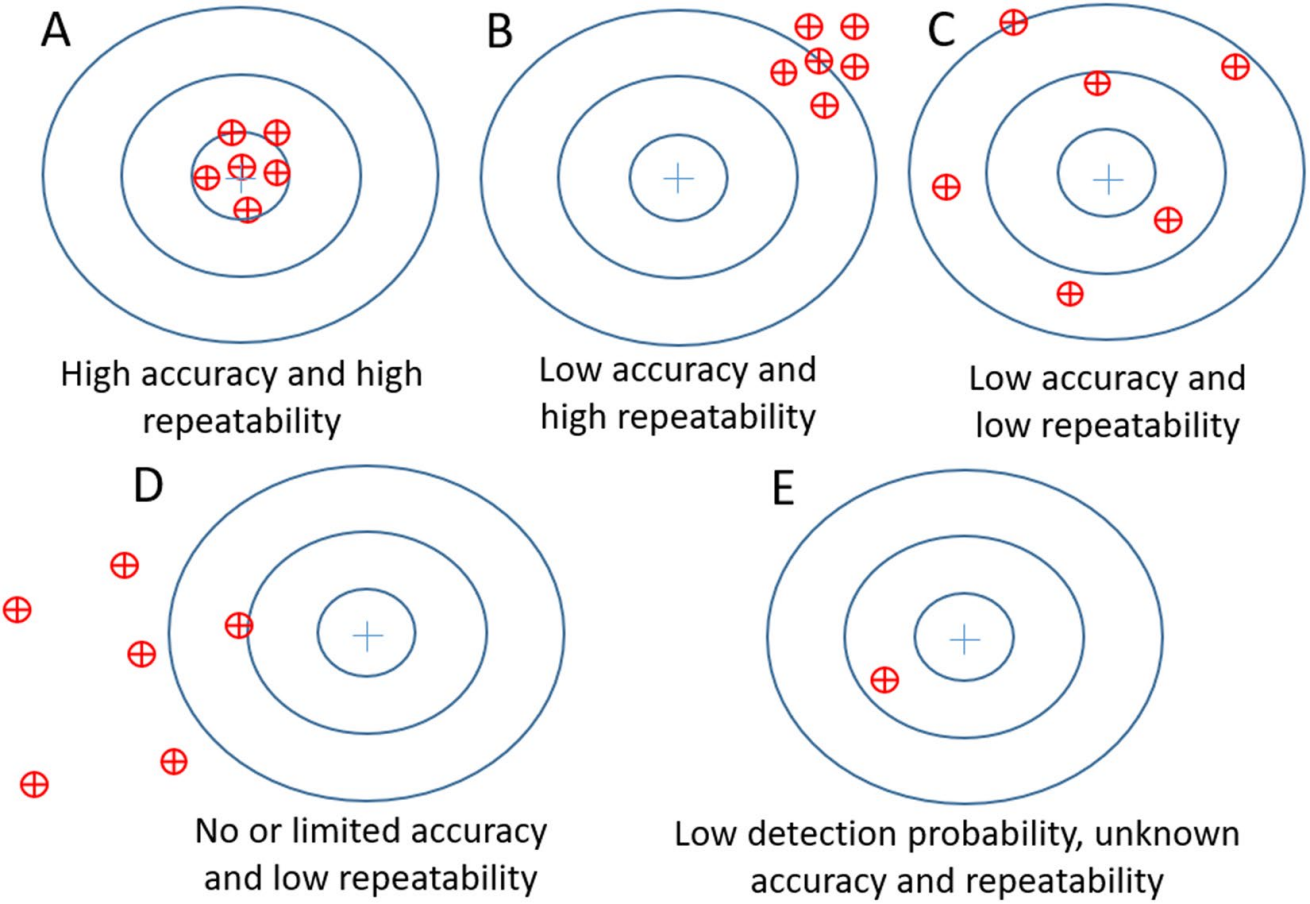
Represent the theoretical variations in eDNA reliability: Blue circles represent a “target” whereby the inner centre circle would represent the higher accuracy. The red circles with crosses in the middle represent “replicate” samples (either natural or technical). Scenario in this figure are as follow: eDNA measurements with high accuracy and high repeatability (**A**), low accuracy and high repeatability (**B**), low accuracy and low repeatability (**C**) and low or limited accuracy and low repeatability. Panel E reflects a case where detection probability is low and hence the accuracy and repeatability of the analysis are unknown.

Detection of eDNA under natural conditions is typically characterized by large variability, due to limited dispersion capacity of eDNA and strong variation in eDNA release and decay, which can also lead to relative low detection probabilities above the LOD^19^. A strategy to improve the accuracy of measurement and reduce the effects of natural variability is to increase the number of replicate samples^20^. Inhibition factors and limitations of the amount of water filtered, can, on the other hand, increase the level of variation seen in any replicate, thereby effecting the assays repeatability^21^. Considering the analysis of an eDNA sample using six qPCR replicates, the efficiency of eDNA detection and quantification in a targeted approach can be separated in to one of five different categories (Fig. 1). High accuracy and high repeatability for example (Fig. 1A), will lead to a high efficiency of eDNA detection and quantification. Low accuracy with high repeatability (Fig. 1B) and low accuracy with low repeatability (Fig. 1C) would, in contrast, lead to a medium efficiency of eDNA detection and poor efficiency of eDNA quantification. Finally, no or very limited accuracy and low repeatability (Fig. 1D) will lead to both poor eDNA detection and quantification. However, depending on the number of positive technical replicates (i.e. qPCR wells for the same sample), eDNA detection can also be obtained with unknown accuracy and repeatability and lead to a low detection probability (Fig. 1E). Besides detection (presence-absence, or species richness), a challenging question is whether we can relate any given species amplicon abundance (i.e. quantification values of the targeted DNA fragment) to the density of said species in its habitat. Because of the low persistence of eDNA particles in aquatic environments, species detection via eDNA allows a reliable survey of species present at any given location^22,23^. However, despite reportedly being a cheaper and more reliable method for species detection than traditional survey methods^24–26^, the vast majority of eDNA studies appear to lack detail in the validation of the methods or assays used. For example, we checked 80 articles (see full list in Annexe. 1) focussing on the eDNA detection of species using barcoding techniques which were published between January 2017 and January 2018 and only 10 mentioned the MIQE Guidelines^1^. Clear method standardisation from field sampling to DNA analysis would greatly improve insights on advantage and disadvantages linked to specific eDNA assays and ultimately increase the transparency and end user confidence.

In this study, we systematically assessed the reliability, detection and quantification limits of different eDNA approaches using the Freshwater Pearl Mussel, *Margaritifera margaritifera* (Linnaeus, 1758) as a target organism. More specifically, our aim was to evaluate accuracy, repeatability and detection probability of two previously designed assays targeting distinct gene regions (COI and 16S) using qPCR^27,28^. Therefore, we first tested the reliability of both assays by establishing standard curves and determining LOD and LOQ following MIQE Guidelines^1,29^. In a second step, we examined the potential of the approaches to serve as an indicator for species abundance. For this purpose, we established and sampled six stable mesocosms with varying mussel densities, and, compared in a double-blind procedure eDNA copy numbers and mussel abundance in mesocosms. In this instance, double blind meant that two teams of researchers were involved in the sampling. The first team collected the water (see methods) while the second team filtered the water without knowing its origin. Furthermore, we did not know the abundance of mussels associated with each sample/mesocosm until all laboratory assessment has been completed. Our results show how the number of water samples per mesocosm (i.e. natural replicates) and the number of qPCR replicates (i.e. technical replicates) are linked to the reliability of quantification. This led us to recommendations for field sampling protocols.

## Results

Both sets of primers and probes were found to be specific in silico. Moreover, the primers from^27^ targeting the 16S gene of *M. margaritifera* were specific against all other mussels tested when using standard PCR (Table 1). The same result was achieved when the probe, (designed in this study), was added for use in qPCR (Table 2). Primers from^28^ aimed at targeting the COI gene of *M. margaritifera* amplified the targeted species and DNA extracted from *Anodonta anatina*, *Truncilla truncata*, and *Cumberlandia monodonta* when run with conventional PCR. However, the addition of the probe (designed in the study by^28^) increased specificity when utilising qPCR and resulted in the single detection of the target species, *M. margaritifera*.

**Table 1:** Results of PCR and qPCR reactions using the primers and probes targeting the COI and 16S gene of *Margaritifera margaritifera* on 12 differents mussel species.

	^27^		^28^	
Target	16S		COI	
Species	PCR	qPCR	PCR	qPCR
*Margaritifera margaritifera*	Amplification	Amplification	Amplification	Amplification
*Margaritifera falcata*	None	None	None	None
*Anodonta anatina*	None	None	Amplification	None
*Unio pictorum*	None	None	None	None
*Anodonta cygnea*	None	None	None	None
*Dreissena rostriformis bugensis*	None	None	None	None
*Dreissena polymorpha*	None	None	None	None
*Corbicula fluminea*	None	None	None	None
*Truncilla truncata*	None	None	Amplification	None
*Quadrula quadrula*	None	None	None	None
*Lampsilis siliquoidea*	None	None	None	None
*Cumberlandia monodonta*	None	None	Amplification	None

**Table 2:** Primers and probes for the detection of environmental DNA traces released by the Freshwater Pearl mussel *Margaritifera margaritifera*.

*Margaritifera margaritifera*			
Target	Primers	Sequence (5′-3′)	Source
COI	Forward	TTGTTGATTCGTGCTGAGTTAGG	^28^
COI	Reverse	GCATGAGCCGTAACAATAACATTG	^28^
COI	Probe	6-FAM- CCTGGTTCTTTGCTGGGT-BHQ-1	^28^
16S	Forward	CAACCCTGGAACCGCTAAAG	^27^
16S	Reverse	GGCTGCGCTCATGTGAATTA	^27^
16S	Probe	6-FAM- TCCAGTTAATCATAGAACTTCATCAAA-BHQ-1	This study

**Figure 2:**
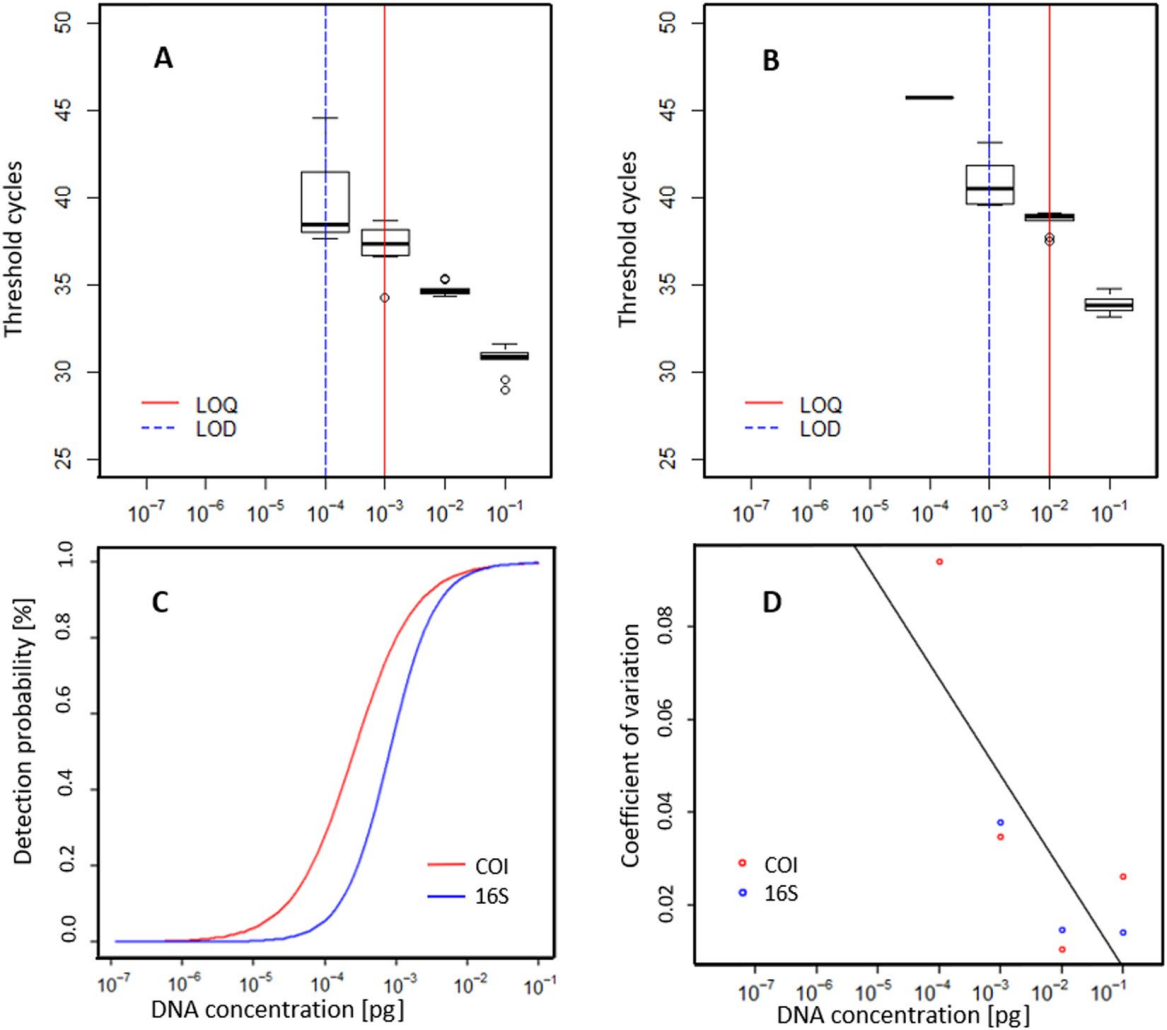
Assessment of standard curves used for quantifying *M. margaritifera* DNA as well as for determining the limit of detection (LOD) and limit of quantification (LOQ) with qPCR targeting the COI (**A**) and the 16S region (**B**). Standard curves were obtained with the same 1:10 dilution series from a starting concentration of 7.8 ng with 10 replicates per concentration. The threshold cycles are representing the minimum number of qPCR amplification cycles leading to positive detection. (**C**) The relationship between the detection probability and DNA concentration for the COI and 16s assays. (**D**) The coefficient of variation of eDNA measurements and its relation to DNA concentration in the standard curve. The black line represents the regression equation for the relationship. Data was pooled from both assays as results did not diverge significantly between methods.

The analysis of the two calibration curves revealed different LOD and LOQ for the two assays (Fig. 2). The COI assay proved to be consistently the more sensitive approach with the LOD and the LOQ falling at 0.78 and 7.8 pg mussel tissue, respectively (Fig. 2A). The 16S assay resulted in a detection of DNA at 0.78 pg mussel tissue. However only one out of 10 replicates was positive showing threshold cycles of 45.74, which does not fulfil the requirements specified in the MIQE guidelines^1^. Hence, the LOD under these rules was 7.8 pg and the LOQ was found to be 78 pg (Fig. 2B).

There was no significant difference (paired t-test, *T* = 0.3, *p* = 0.79) between the accuracy of the two assays, or between the natural replicates (ANOVA, *p* = 0.12). Further, the repeatability (indicated by the *r*^2^ of the calibration curves), was quite similar for the two assays (COI: Adjusted *r*^2^ = 0.985; 16S: Adjusted *r*^2^ = 0.974). Further, the detection probability of both assays decreased with the dilution rate of tissue samples (16S assay *r*² = 0.88 and COI assay *r*² = 0.8488). Likewise, the accuracy of both assays (which was represented by the CV of technical replicates), decreased with the dilution of sample DNA (log(y) = −0.23 log(x) − 4.86; Adjusted *r*^2^ = 0.52; *p* = 0.04). At the LOD, the CV was 0.09 and 0.04 for the COI and 16S assays, respectively (Fig. 2D).

In our mesocosm experiment, environmental conditions were as follows: Temperature was 11.96 ± 0.054 °C, pressure 15.1 ± 0.018 PSI, turbidity 0.2125 FNU ± 0.126, pH 7.032 ± 0.005, rugged DO 10.06 ± 0.025 mm L^−1^ and conductivity 45.564 ± 0.212 µS cm^−1^. Flow rates were kept constant within mesocosms and only varied slightly between them (0.75 to 1.03 Ls^−1^). Water samples from Lake Windermere (before and after the facilities internal filtration process), were all found to be negative for the presence of eDNA from *M. margaritifera* using both assays, showing that the eDNA from the mesocosms is not being recirculated from the lake.

**Figure 3:**
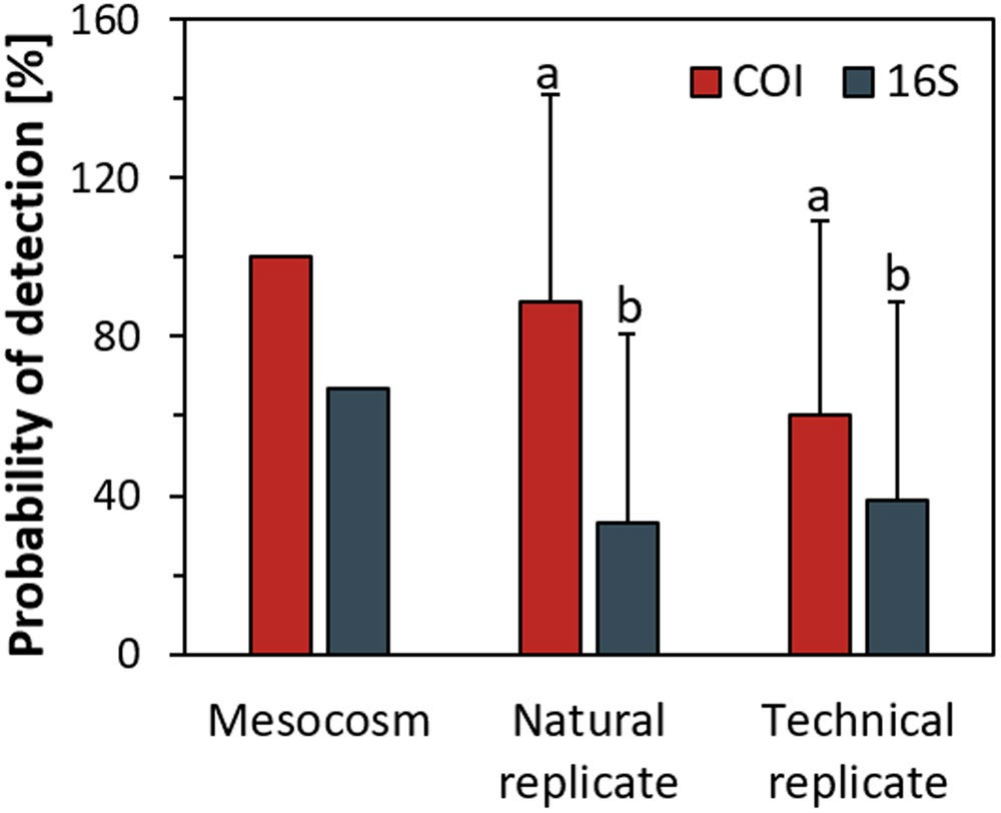
Probability of detecting eDNA in mesocosms (n = 6), natural replicates (n = 18) and technical replicates (n = 108) for the COI and the 16s assay. Error bars represent standard deviation and letters a, b relates to significant differences between assay at the natural and technical replicate level assessed by paired t-tests.

The COI assay resulted in positive DNA signals in 100% of the mesocosms. The 16S assay, on the other hand, detected mussel DNA in only four out of six mesocosms and showed lower detection probability at the level of natural and technical replicates (Fig. 3). After this result, it would have been preferable to test the DNA of the six different populations to assess if there were various genetically distinct haplotypes present (i.e. did two of these populations have point mutations in the conserved 16S region where the assay targeted). However, this was not possible (at the current time) as these animals are part of a breeding program and therefore tissue collection was avoided. That said, this should be considered in future studies. Furthermore, the detection probability for the 16S assay was higher for technical replicates than for natural replicates, a result due to the absence of eDNA detection in several of the mesocosms. For the above reasons, we decided to focus further statistical analysis only on the COI assay.

**Figure 4:**
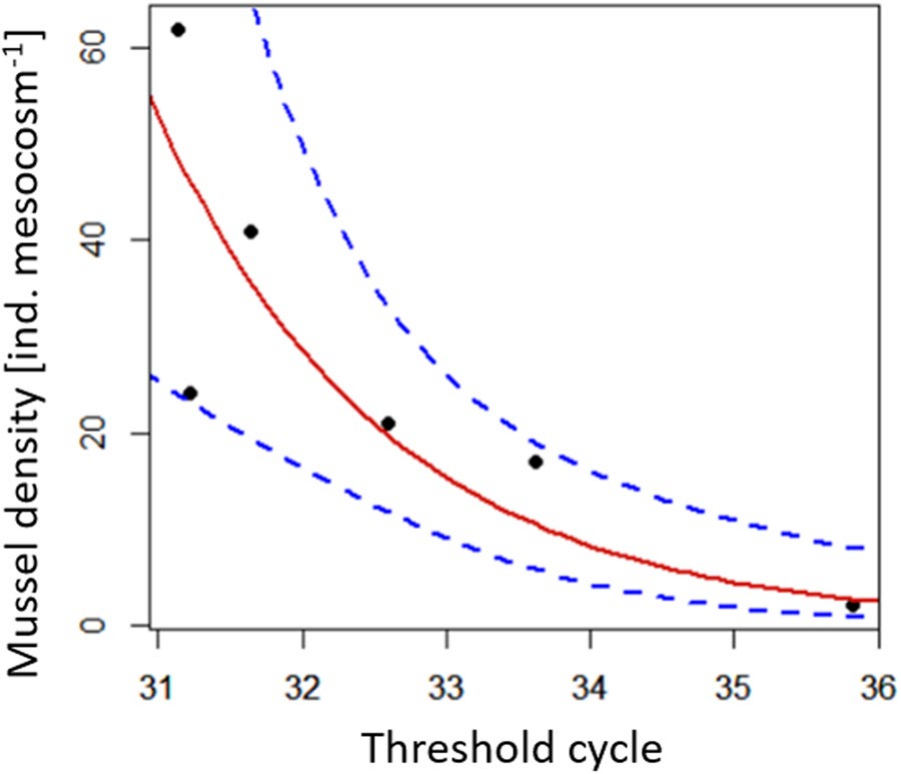
Relationship between the number of individuals of *M. margaritifera* present per mesocosm and the threshold cycles of eDNA detection of mesocosm samples (points represent average of natural replicates) generated by the COI assay. Red line represents the regression equation of the relationship, doted blue lines indicate the confidence interval.

**Figure 5:**
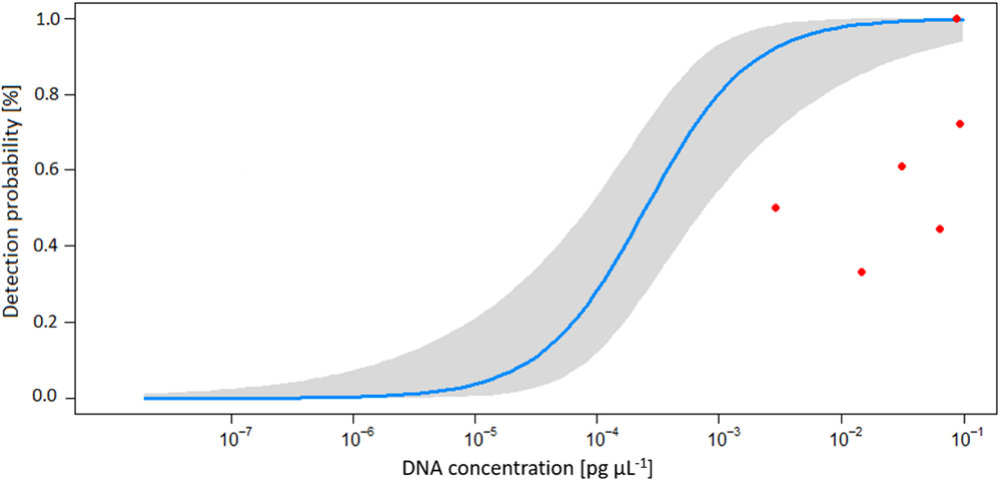
Change in detection probability with increasing DNA concentration in samples analysed with the COI assay. The blue curve and the grey-shaded area reflect the regression, established by analysing the standard curve, and its confidence interval. Red dots represent data from the mesocosm experiment. We used the standard curve for eDNA quantification (Fig. 2A) to convert threshold cycles of detection measured in mesocosm samples to DNA concentrations.

A significant negative correlation was found between, mussel density and the logged threshold cycles of detection in the mesocosm experiment (y = −1,422 × +36,842, *r*^2^ = 0.88, *p* < 0.01), hence the threshold cycles increased logarithmically with decreasing mussel densities in the mesocosms (Fig. 4). However, neither detection probability nor the CV of the mesocosms were significantly related to the threshold cycle of detection and mussel densities in the mesocosms (*p* > 0.19). Consequently, five out of six of the mesocosms ranged outside the confidence interval of the relation between detection probability and the number of threshold cycles leading to detection, which was established based on data from the standard curve (Fig. 5).

**Figure 6:**
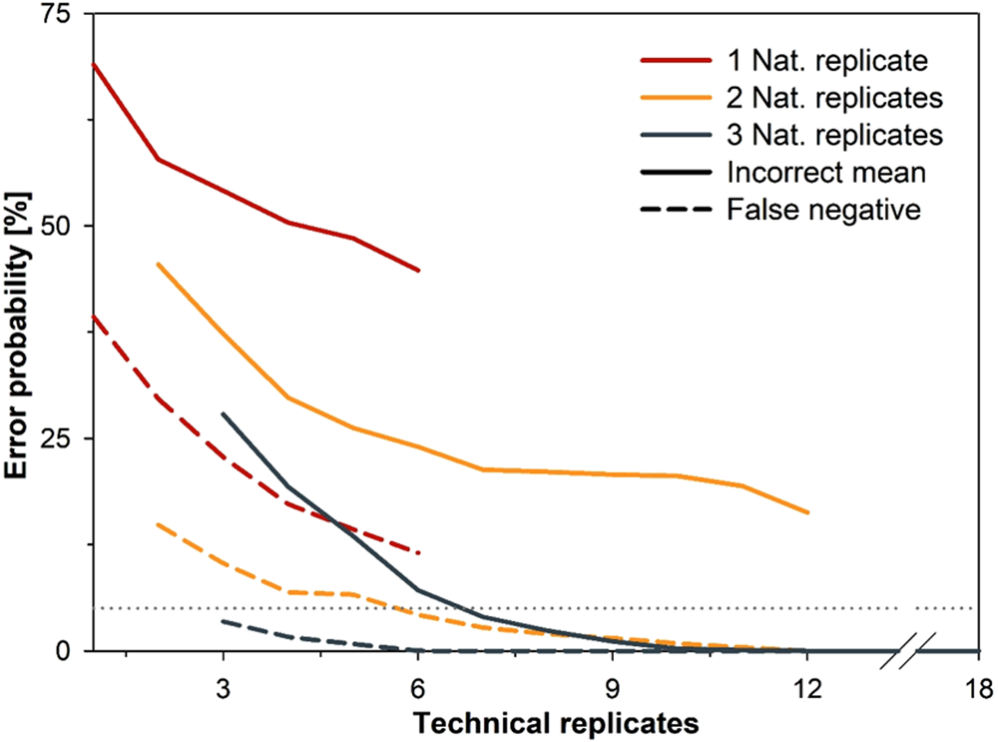
Impact of sampling design on the reliability of eDNA measurements. Results were generated by subsampling data from the mesocosm experiment in a bootstrap approach to reveal the change of the probability attaining false negatives or incorrect means, i.e. means that range outside the confidence interval of the original relationship, with increases in the number of natural and technical replicates. Statistical simulations were based on results of the COI assay.

We were also interested to test if natural replicates taken from the same mesocosm showed a significant difference in their threshold cycle of detection. In five out of the six mesocosms, a sufficient number of replicates yielded positive results to allow for this test to be conducted. In two of these five mesocosms, natural replicates showed significantly different results (p < 0.03) with regard to their threshold cycle of detection. With mean difference of 1.1 and 1.5 threshold cycles. A bootstrapping approach (to assess the effect of reduced replicate numbers on the reliability of measurements), revealed that a high number of replicates was required to ensure method sensitivity and accurate eDNA quantification (Fig. 6). The reduction of natural replicates had thereby a more negative impact on method reliability than the reduction of technical replicates, highlighting the importance of taking multiple water samples at the same field site.

## Discussion

In this study, we assessed the reliability of two different assays targeting the COI and 16S gene, which have been previously designed for the assessment of environmental DNA of the endangered Freshwater Pearl Mussel, *M. margaritifera*. Originally, the COI assay showed non-specificity during PCR, but in combination with a species-specific probe during qPCR, the specificity of this assay substantially increased and only targeted *M. margaritifera*^28^. In contrast, the 16S assay showed specificity during both conventional PCR and qPCR. Applying both assays on the eDNA samples of the mesocosm experiment revealed that the efficiency of COI outperformed the 16S assay in terms of the LOD and LOQ, whereas the threshold cycles appeared to be lower for the same dilution of standard samples. Despite being specific to *M. margaritifera*, detection of eDNA using the 16S assay failed in two out of the six mesocosms, where known mussels were present. These findings reemphasize the call by^30^ about the importance of rigorous *ex-situ* tests (under controlled experimental conditions) in order to validate assays before the use of eDNA in the field. Although we applied already published primers and qPCR assays, method validation against MIQE guidelines and an additional test under controlled experimental conditions was necessary to select the most efficient and reliable primers/probe for qPCR quality^1,29^. Furthermore, the size of both markers could also explain the difference in the efficiency of eDNA detection. As specified in various studies^31,32^, larger fragments of DNA (172 bp for 16S) degrade more rapidly than small fragments (83 bp for COI), and are therefore less abundant in natural environments^33,34^.

A key finding of our study was that method validation and obtained sensitivity of primers and qPCR under lab-settings can differ largely from results attained in more natural environmental conditions. High concentration of mussel DNA in the standard dilution led to high detectability and high efficiency of eDNA detection and quantification compared to high dilution standards. As described in^29^, qPCR detection had minor variation among replicates of samples. However, instrumental response shows poor reproducibility at low eDNA concentrations which is typical of eDNA samples^29^. Here, we found that the same results were achievable (when exploring the standard curves) (Fig. 5) using both assays assessed. Surprisingly, when a high number of mussels were present in a given mesocosm, we did not observe a higher detection probability amongst replicates than in mesocosms with low numbers of mussels. Furthermore, in our study the LOD for eDNA from *M. margaritifera* (10^−4^ ng) was similar to that shown in^28^ i.e. quite high when compared to other animal groups^35,36^. For example, for the invasive crayfish *Procambarus clarkii* (Girard, 1852) and for the endangered newt *Triturus cristatus* (Laurenti, 1768), LODs of 10^−7^ ng and lower were reported^35,36^. A relative high LOD represents a potential limiting factor for detecting eDNA from mussel species with low abundance in the field. However, operating relatively close to the LOD in this study, allowed us to assess the reliability of eDNA detection and quantification under stress-conditions, which are likely frequently encountered during in-*situ* eDNA assessments. There is also a slight possibility that PCR inhibitors were affecting the eDNA detection in this study. Although we did not test for PCR inhibition within our samples, we believe inhibition (if present) would be low for three main reasons. The water entering each mesocosms was filtered (through a 20-micron Hydrotech Drumfilter HDF800-series), in the absence of any other physical or chemical treatment. As the eDNA samples were taken within centimetres of the mussels, this is unlikely to play an important factor here. Third and finally, inhibition has also been shown to be driven by compounds produced via various biological processes of phytoplankton and plant matter for example^21^, the filtered water would have removed the vast majority of these compounds.

Interestingly, a positive relationship between eDNA quantification and the mussels density in mesocosms was illustrated, highlighting similar levels of repeatability as seen in various studies on other organisms^8,12,14,15^. In our study, however, the relationship between the number of mussels present in the mesocosm experiment and the eDNA quantification was non-linear. Therefore, although we have found that quantification appears to be possible for *M. margaritifera* using eDNA, further studies still need to be conducted in order to assess the effects of various environmental variables on species quantification. In fact, one of the key objectives in any eDNA study should be the exploration of factors that increase and decrease the eDNA sheading rate per species and in this instance, we have highlighted that density is certainly one to take into account. Exploring the effect of biological and environmental factors including temperature, pH, flow rate and sedimentation (as in^37–39^ for example) will improve our understanding of the variability of eDNA sheading rates under natural conditions. Furthermore, the method of filtration could also be explored in more detail and may be important in optimising the assay for management and mitigation applications. Here we utilised enclosed Sterivex filters which were highlighted by^40^ as being desirable. However, these remain costly and the use of cellulose nitrate filters has been recently proposed to be better than (or at least equal to) the Sterivex method^41^. The reduced cost of these filters means they should certainly be considered for use in future studies.

Finally, we assessed how to improve the efficiency of eDNA sampling strategies for *M. margaritifera*. Our statistical modelling approach revealed that the collection of three natural replicates per field location is required to ensure a high reliability of eDNA detection and quantification. However, we recommend an even higher number of natural replicates should be collected (four to six for example), as this will likely further increase the repeatability and accuracy of species quantification in the field and having more than three allows for the possible failure or poor extraction of DNA from any one given sample. On the other hand, the number of technical replicates could be reduced because the analyses of four technical replicates (per natural replicate), was sufficient to reduce the expected error probability below 1% (Fig. 6). Based on these findings, it is also recommended to use standard dilutions on each PCR plate, both as a positive control but also for estimating the LOD of the analysed samples^29,42,43^. Thereby, the MIQE guidelines can be used for assessing the efficiency of any newly developed assay and should be a minimum standard for all eDNA studies moving forward^1,29^.

In conclusion, our findings reveal that different methodological aspects influence the reliability of eDNA assays at various levels. Method selection was mostly dependant on detection probability and the LOD, as accuracy and repeatability were similar for both assays assessed in this study. However, species quantification mostly relied on repeatability, despite the use of three natural replicates from mesocosms scattered around regression predictions. Finally, method efficiency represented by the minimum effort for obtaining robust results was dependant on accuracy and detection probability of measurements. These factors were proven to be critical because of the observed high variability between natural replicates and the detection probability of ~50% as this is clearly above the LOD.

## Materials and Methods

### Study species and system

The target species of our study was the rare and protected Freshwater Pearl Mussel (*M. margaritifera*), a large (~14 cm) bivalve with a maximum life span of over 100 years and a generation time of 30 years^28,44^. While it was once a dominant and functionally important species, it has since declined across the majority of its former range by upwards of 62%. The species was therefore classified as endangered throughout Europe in 1996^44^. Application of eDNA approaches on mussel species are in principle characterised by a relative low sensitivity^28^ and hence investigations with *M. margaritifera* represent a suitable yard stick to assess the reliability of eDNA based species quantification.

The experimental part of the study was performed at the Freshwater Biological Association (FBA) Ark station in Windermere; a unique facility which has been holding this critically endangered species under controlled conditions for the past 10 years. Currently, 167 adult *M. margaritifera* (from six different river populations) are housed in six independently maintained mesocosms. The experimental mesocosms are circular, 1.6 m³ in size and continuously supplied with water filtered through a 20-micron Hydrotech Drumfilter HDF800-series. The water is obtained directly from Lake Windermere, and no other physical or chemical treatment is utilised. Prior to the experiment, water samples from before and after the facility filtration process were tested in order to ensure the absence of targeted DNA in the water entering each mesocosm. Additionally, we measured various physio-chemical water parameters to confirm the match with environmental conditions in natural breeding sites.

### Sampling and PCR protocols

Tissue samples (*n* = 12) from Bivalve species: *Margaritifera margaritifera*, *Margaritifera falcata* (Gould, 1850), *Anodonta anatina* (Linnaeus, 1758), *Anodonta cygnea* (Linnaeus, 1758), *Unio pictorum* (Linnaeus, 1758), *Dreissena rostriformis bugensis* (Andrusov, 1897), *Dreissena polymorpha* (Pallas, 1771), *Corbicula fluminea* (Müller, 1774), *Truncilla truncata* (Rafinesque, 1820), *Quadrula quadrula* (Rafinesque, 1820), *Lampsilis siliquoidea* (Barnes, 1823) and *Cumberlandia monodonta* (Say, 1829) were collected to establish standard curves and the specificity of the approach. Tissue samples were preserved in absolute ethanol and kept at −80 °C until extraction (see below). Water samples (for eDNA analysis) were taken on the 1^st^ November 2017. From each mesocosm, we collected three 1 L water samples with a sterile polypropylene ladle from the water surface. Samples were collected in a sterile plastic bag (Whirl-Pak® 1242 ml Stand-Up Bag Merck®, Darmstadt, Germany) and filtered with a 50-mL syringe (sterile Luer-Lock™ BD Plastipak™, Ireland) through a sterile 0.45 µm Sterivex™ HV filter (Sterivex™ filter unit, HV with luer-lock outlet, Merck®, Millipore®, Germany)^43^. To avoid contamination, we used disposable nitrile gloves during the sampling process and replaced them between each sample. All filters were stored in 50 mL tubes (Falcon™ 50 ml Conical Centrifuge Tube, Fisher Scientific, Ottawa, Canada) at −80 °C before extraction.

From both the water and tissue samples, DNA was extracted using the Qiagen DNeasy® Blood and Tissue Kit following manufacturers’ guidelines. For the water samples, a slight modification to these were applied following methods outlined in^43^. Control samples, i.e. water samples without traces of *M. margaritifera* DNA and separate samples consisting of ddH_2_O were also extracted as above. Pipettes and tube holders were disinfected and regularly decontaminated under UV-treatment. All other lab equipment and surfaces were regularly disinfected using 10% bleach solution and ethanol before the analysis.

PCR amplification was performed on a Gen Amp® PCR System 9700 (Applied Biosystem) by using two sets of pre-designed species-specific primers^27,28^. The set designed by^28^ targeted the mitochondrial cytochrome oxidase subunit I gene (COI) while the set designed by^27^ targeted the DNA sequence of the 16S rRNA subunit (Table 2). PCR reactions were performed in a 25 µL total volume with 12.5 µL of 2x PCRBIO Ultra Mix Red (PCRBIOSYSTEMS), 1 µL of each primer (10 µM), 9.5 µL of ddH_2_O and 1 µL of DNA template. For the COI primers, the PCR protocol followed that outlined in^28^ with slight modifications. Briefly, an initial warming step at 50 °C for 2 min and denaturation at 95 °C for 10 min, was followed by 35 cycles 95 °C for 15 s and 60 °C for 1 min. For the 16S primer, the PCR protocol followed^27^, with slight modifications. These included, an initial denaturation at 95 °C for 15 s, followed by 35 cycles of 95 °C for 15 s, 60 °C for 10 s and 72 °C for 20 s. Products from PCR were visualized on 2% agarose gel stained with GelRed™. qPCR programmes were similar to PCR programmes but were performed with 55 instead of 35 cycles. For this study we designed a complementary probe (6-FAM- TCCAGTTAATCATAGAACTTCATCAAA-BHQ-1) to work with the 16S primers. This was done with Geneious Pro R10 software http://www.geneious.com; and as in^45^. The probe was assessed for specificity against DNA sequences retrieved from tissue samples of the targeted species and from closely related species and other mussel species that potentially can live in the same ecosystem as *M. margaritifera* (Table 1 – extracted in this study) along with other sequences retrieved from NCBI (i.e. National Centre for Biotechnology Information) database (https://www.ncbi.nlm.nih.gov/). For the qPCR assay targeting the COI, the probe described by^28^ was used. The total amplicon size (including primers) was 83 bp for COI and 172 bp for 16S. Specificity of primers and probes were assessed in silico using Geneious Pro R10 software and *in vitro* by PCR and qPCR. Primers and probes were tested against tissues of eleven other mussel species (See Table 1). qPCR assays were performed in a final volume of 25 µl using 12.5 µl of PrecisionPlus qPCR Master Mix with ROX (Primer Design, UK), 1 µl of each primer (10 µM), 1 µl of the corresponding probe (2.5 µM), 6.5 µl of ddH_2_O and 3 µl of extracted DNA on an ABI StepOnePlus™ Real-Time PCR (Applied Biosystems).

First, we established calibration curves by analysing a 1:10 dilution series of the DNA from tissue samples of *M. margaritifera* (7.8 ng/µl, Nanodrop 2000 Spectrophotometer, Thermofisher Scientific). This dilution series ranged from 10^−1^ to 10^−7^. We ran 10 technical replicates for each dilution step in order to assess the LOD and LOQ^1,29^. The LOD was defined as the last standard dilution when the targeted DNA was detected and quantified in at least one qPCR replicate with a threshold cycle under 45. The LOQ (and therefore the sensitivity of the assay) was defined as the last standard dilution when the targeted DNA was detected and quantified in at least 90% of replicates of the standard dilution with a threshold cycle under 45. Each PCR and qPCR, with DNA extracted from tissues, was run in duplicate and was replicated at least two times. At least two negative controls were included in each run. Then, the DNA extracts obtained from all water samples from the mesocosm-experiment were analysed in six technical replicates in qPCR with at least four negative controls and two replicates of the dilution series from 10^−1^ to 10^−4^ as positive controls.

### Statistical analysis

Standard dilution series obtained for the COI and 16S-based assays were used for determining the LOD and LOQ^29,35^. Linear regressions between dilution factor of tissue samples and the DNA concentration (i.e. means of technical replicates used) were established and *r*^2^ of the regression was evaluated as a measure of repeatability of qualification. Further, we examined the relationship between; (a) detection probability, i.e. the percentage of technical replicates that lead to a positive result, and (b) the coefficient of variation (CV, calculated as standard deviation divided by mean) of technical replicates within a sample to the dilution rate of tissue samples in a regression analysis.

While experimental samples from mesocosms were analysed with both genetic assays, the 16S assay showed a lower detection probability than the COI (see results), therefore, further analysis was only conducted using the COI assay. The relationships between eDNA detection and mussel density in mesocosms was assessed in an ordinary least square regression analysis where *r*^2^ representing the repeatability of quantification. The effect of mussel densities on detection probability and accuracy (i.e. CV within natural replicates) was likewise evaluated in linear regressions. The importance of natural variability, represented by the variability between natural replicates, was analysed using a one-way ANOVA. Regression analyses were tested for non-linearities by establishing separate regression models for non- and log-transformed data and comparing the models fit using Akaike Information Criterion (AIC) and log-transformed data as necessary. Residuals were analysed, and no pattern or autocorrelation was found. Homogeneity of variance was evaluated using a Bartlett test prior to ANOVAs and if necessary measurements were transformed to achieve homoscedasticity. If transformations did not culminate in homogeneity of variances, a pairwise Wilcox test was used instead of ANOVA.

Finally, we investigated the effect of the number of technical and natural replicates on the reliability of eDNA measurements using a boot-strap approach and the results of the mesocosm experiment as a data pool. For a given combination of natural and technical replicates, data from each mesocosm was subsampled 10,000 times and the mean eDNA concentration for each subsample was calculated. Based on these simulations, we were able to determine; (i) the mean probability of false negative detection across all mesocosms and (ii) the mean probability to achieve an incorrect result. A “false negative” was thereby defined as a case when DNA was present in a mesocosm (as in all cases in this study), but undetected by the assay. An “incorrect result” on the other hand, was defined as a case when the mean eDNA concentration ranged outside the confidence interval of the regression between mussel density and eDNA concentration. We repeated this procedure for all possible combinations of 1–3 natural and 1–18 technical replicates. All statistical analyses and models were performed with R version 3.4.1^46^.

## Supplementary information

Supplementary Information

Supplementary dataset
