# Supplementary material for: Influence of accuracy, repeatability and detection probability in the reliability of species-specific eDNA based approaches
Source: Sci Rep. 2019 Jan 24;9:580. doi: 10.1038/s41598-018-37001-y (PMC6345946; doi:10.1038/s41598-018-37001-y)
Supplement: Supplementary file 1 — Supplementary Information [file 41598_2018_37001_MOESM1_ESM.pdf]

Influence of accuracy, repeatability and detection probability in the reliability of species-specific eDNA based approaches

Quentin Mauvisseau [1 2], Alfred Burian [1], Ceri Gibson [3], Rein Brys [4], Andrew Ramsey [1], Michael Sweet [1]

[1] Aquatic Research Facility, Environmental Sustainability Research Centre, University of Derby, Derby, DE22 1GB, UK

[2] Surescreen Scientifics Ltd, Morley Retreat, Church Lane, Morley, DE7 6DE, UK

[3] Freshwater Biological Association, Ferry Landing, Far Sawrey, Ambleside, Cumbria LA22 0LP, UK

[4] Research Institute for Nature and Forest, Gaverstraat 4, 9500 Geraardsbergen, Belgium

Corresponding author: q.mauvisseau@derby.ac.uk

Sensitivity: Internal

Annexe 1: List of articles using eDNA barcoding techniques refering or not to the MIQE Guidelines

Abreviated references	Reference to the MIQE Guidelines	Full article references
Williams et al. 2018	No	Williams, K. E., Huyvaert, K. P., Vercauteren, K. C., Davis, A. J., & Piaggio, A. J. (2018). Detection and persistence of environmental DNA from an invasive, terrestrial mammal. Ecology and Evolution, 8(1), 688–695. doi:10.1002/ece3.3698
Takahashi et al. 2018	No	Takahashi, M. K., Meyer, M. J., Mcphee, C., Gaston, J. R., Venesky, M. D., & Case, B. F. (2018). Seasonal and diel signature of eastern hellbender environmental DNA: Temporal Signature of Hellbender eDNA. The Journal of Wildlife Management, 82(1), 217–225. doi:10.1002/jwmg.21349
Seymour et al. 2018	No	Seymour, M., Durance, I., Cosby, B. J., Ransom-Jones, E., Deiner, K., Ormerod, S. J., … Creer, S. (2018). Acidity promotes degradation of multi-species environmental DNA in lotic mesocosms. Communications Biology, 1(1). doi:10.1038/s42003-017-0005-3
Nevers et al. 2018	No	Nevers, M. B., Byappanahalli, M. N., Morris, C. C., Shively, D., Przybyla-Kelly, K., Spoljaric, A. M., … Roseman, E. F. (2018). Environmental DNA (eDNA): A tool for quantifying the abundant but elusive round goby (Neogobius melanostomus). *PloS One* , *13* (1), e0191720.
Geerts et al. 2018	No	Geerts, A. N., Boets, P., Van den Heede, S., Goethals, P., & Van der heyden, C. (2018). A search for standardized protocols to detect alien invasive crayfish based on environmental DNA (eDNA): A lab and field evaluation. Ecological Indicators, 84, 564–572. doi:10.1016/j.ecolind.2017.08.068
Cowart et al. 2018	No	Cowart, D. A., Renshaw, M. A., Gantz, C. A., Umek, J., Chandra, S., Egan, S. P., … Larson11, E. R. (2018). Development and field validation of an environmental DNA (eDNA) assay for invasive clams of the genus Corbicula. *Management of Biological Invasions* .
Buxton et al. 2018	No	Buxton, A. S., Groombridge, J. J., & Griffiths, R. A. (2018). Seasonal variation in environmental DNA detection in sediment and water samples. PLOS ONE, 13(1), e0191737. doi:10.1371/journal.pone.0191737

Yamanaka et al. 2017	No	Yamanaka, H., Minamoto, T., Matsuura, J., Sakurai, S., Tsuji, S., Motozawa, H., … Kondo, A. (2017). A simple method for preserving environmental DNA in water samples at ambient temperature by addition of cationic surfactant. Limnology, 18(2), 233–241. doi:10.1007/s10201-016-0508-5
Xia et al. 2017	No	Xia, Z., Zhan, A., Gao, Y., Zhang, L., Haffner, G. D., & MacIsaac, H. J. (2017). Early detection of a highly invasive bivalve based on environmental DNA (eDNA). Biological Invasions. doi:10.1007/s10530-017-1545-7
Wittwer et al. 2017	No	Wittwer, C., Stoll, S., Strand, D., Vrålstad, T., Nowak, C., & Thines, M. (2017). eDNA-based crayfish plague monitoring is superior to conventional trap-based assessments in year-round detection probability. Hydrobiologia. doi:10.1007/s10750-017-3408-8
Wilson et al. 2017	No	Wilson, J.-J., Sing, K.-W., Chen, P.-N., & Zieritz, A. (2017). Tracking the southern river terrapin (Batagur affinis) through environmental DNA: prospects and challenges. Mitochondrial DNA Part A, 1–5. doi:10.1080/24701394.2017.1373109
Williams et al. 2017	No	Williams, M. R., Stedtfeld, R. D., Engle, C., Salach, P., Fakher, U., Stedtfeld, T., … Hashsham, S. A. (2017). Isothermal amplification of environmental DNA (eDNA) for direct field-based monitoring and laboratory confirmation of Dreissena sp. PLOS ONE, 12(10), e0186462. doi:10.1371/journal.pone.0186462
Williams et al. 2017	No	Williams, K. E., Huyvaert, K. P., Vercauteren, K. C., Davis, A. J., & Piaggio, A. J. (2017). Detection and persistence of environmental DNA from an invasive, terrestrial mammal. Ecology and Evolution. doi:10.1002/ece3.3698
Williams et al. 2017	No	Williams, K. E., Huyvaert, K. P., & Piaggio, A. J. (2017). Clearing muddied waters: Capture of environmental DNA from turbid waters. *PloS One* , *12* (7), e0179282.
Weltz et al. 2017	No	Weltz, K., Lyle, J. M., Ovenden, J., Morgan, J. A., Moreno, D. A., & Semmens, J. M. (2017). Application of environmental DNA to detect an endangered marine skate species in the wild. *PloS One* , *12* (6), e0178124.
Vörös et al. 2017	No	Vörös, J., Márton, O., Schmidt, B. R., Gál, J. T., & Jelić, D. (2017). Surveying Europe's Only Cave-Dwelling Chordate Species (Proteus anguinus) Using Environmental DNA. PLOS ONE, 12(1), e0170945. doi:10.1371/journal.pone.0170945

Ulibarri et al. 2017	No	Ulibarri, R. M., Bonar, S. A., Rees, C., Amberg, J., Ladell, B., & Jackson, C. (2017). Comparing Efficiency of American Fisheries Society Standard Snorkeling Techniques to Environmental DNA Sampling Techniques. North American Journal of Fisheries Management, 37(3), 644–651. doi:10.1080/02755947.2017.1306005
Uchii et al. 2017	No	Uchii, K., Doi, H., Yamanaka, H., & Minamoto, T. (2017). Distinct seasonal migration patterns of Japanese native and non-native genotypes of common carp estimated by environmental DNA. Ecology and Evolution. doi:10.1002/ece3.3346
Tsuji et al. 2017	No	Tsuji, S., Yamanaka, H., & Minamoto, T. (2017). Effects of water pH and proteinase K treatment on the yield of environmental DNA from water samples. Limnology, 18(1), 1–7. doi:10.1007/s10201-016-0483-x
Tsuji et al. 2017	No	Tsuji, S., Ushio, M., Sakurai, S., Minamoto, T., & Yamanaka, H. (2017). Water temperature-dependent degradation of environmental DNA and its relation to bacterial abundance. *PloS One* , *12* (4), e0176608.
Torresdal et al. 2017	No	Torresdal, J. D., Farrell, A. D., & Goldberg, C. S. (2017). Environmental DNA Detection of the Golden Tree Frog (Phytotriades auratus) in Bromeliads. *PLOS ONE* , *12* (1), e0168787.
Takahashi et al. 2017	No	Takahashi, M. K., Meyer, M. J., Mcphee, C., Gaston, J. R., Venesky, M. D., & Case, B. F. (2017). Seasonal and diel signature of eastern hellbender environmental DNA: Temporal Signature of Hellbender eDNA. The Journal of Wildlife Management, 82(1), 217–225. doi:10.1002/jwmg.21349
Strobel et al. 2017	No	Strobel, B., Laramie, M. B., & Pilliod, D. S. (2017). Exploring the use of Environmental DNA to Determine the Species of Salmon redds. North American Journal of Fisheries Management. doi:10.1080/02755947.2017.1335254
Stoeckle et al. 2017	No	Stoeckle, B. C., Beggel, S., Cerwenka, A. F., Motivans, E., Kuehn, R., & Geist, J. (2017). A systematic approach to evaluate the influence of environmental conditions on eDNA detection success in aquatic ecosystems. *PloS One* , *12* (12), e0189119.
Stewart et al. 2017	No	Stewart, K., Ma, H., Zheng, J., & Zhao, J. (2017). Using environmental DNA to assess population-wide spatiotemporal reserve use. Conservation Biology. doi:10.1111/cobi.12910

Stephen et al. 2017	No	Stephen L. Klobucar, Torrey W. Rodgers, & Phaedra Budy. (2017). At the forefront: evidence of the applicability of using environmental DNA 1 to quantify the abundance of fish populations in natural lentic waters with additional sampling considerations. *Canadian Journal of Fisheries and Aquatic Sciences* .
Spens et al. 2017	Yes	Spens, J., Evans, A. R., Halfmaerten, D., Knudsen, S. W., Sengupta, M. E., Mak, S. S. T., … Hellstr?m, M. (2017). Comparison of capture and storage methods for aqueous macrobial eDNA using an optimized extraction protocol: advantage of enclosed filter. Methods in Ecology and Evolution, 8(5), 635–645. doi:10.1111/2041-210X.12683
Song et al. 2017	No	Song, J. W., Small, M. J., & Casman, E. A. (2017). Making sense of the noise: The effect of hydrology on silver carp eDNA detection in the Chicago area waterway system. Science of The Total Environment, 605–606, 713–720. doi:10.1016/j.scitotenv.2017.06.255
Shogren et al. 2017	No	Shogren, A. J., Tank, J. L., Andruszkiewicz, E., Olds, B., Mahon, A. R., Jerde, C. L., & Bolster, D. (2017). Controls on eDNA movement in streams: Transport, Retention, and Resuspension. Scientific Reports, 7(1). doi:10.1038/s41598-017-05223-1
Serrao et al. 2017	Yes	Serrao, N. R., Reid, S. M., & Wilson, C. C. (2017). Establishing detection thresholds for environmental DNA using receiver operator characteristic (ROC) curves. Conservation Genetics Resources. doi:10.1007/s12686-017-0817-y
Sansom and Sassoubre 2017	Yes	Sansom, B. J., & Sassoubre, L. M. (2017). Environmental DNA (eDNA) Shedding and Decay Rates to Model Freshwater Mussel eDNA Transport in a River. Environmental Science & Technology, 51(24), 14244–14253. doi:10.1021/acs.est.7b05199
Sakata et al. 2017	No	Sakata, M. K., Maki, N., Sugiyama, H., & Minamoto, T. (2017). Identifying a breeding habitat of a critically endangered fish, Acheilognathus typus, in a natural river in Japan. The Science of Nature, 104(11–12). doi:10.1007/s00114-017-1521-1
Roy et al. 2017	No	Roy, M., Belliveau, V., Mandrak, N. E., & Gagné, N. (2017). Development of environmental DNA (eDNA) methods for detecting high-risk freshwater fishes in live trade in Canada. Biological Invasions. doi:10.1007/s10530-017-1532-z

Rodgers et al. 2017	No	Rodgers, T. W., Olson, J. R., Klobucar, S. L., & Mock, K. E. (2017). Quantitative PCR assays for detection of five arctic fish species: Lota lota, Cottus cognatus, Salvelinus alpinus, Salvelinus malma, and Thymallus arcticus from environmental DNA. Conservation Genetics Resources. doi:10.1007/s12686-017-0883-1
Rees et al. 2017	No	Rees, H. C., Baker, C. A., Gardner, D. S., Maddison, B. C., & Gough, K. C. (2017). The detection of great crested newts year round via environmental DNA analysis. BMC Research Notes, 10(1). doi:10.1186/s13104-017-2657-y
Pitt et al. 2017	No	Pitt, A. L., Shinskie, J. L., Tavano, J. J., Hartzell, S. M., Delahunty, T., & Spear, S. F. (2017). Decline of a giant salamander assessed with historical records, environmental DNA and multi-scale habitat data. Freshwater Biology, 62(6), 967–976. doi:10.1111/fwb.12917
Piggott et al. 2017	No	Piggott, M. P. (2017). An environmental DNA assay for detecting Macquarie perch, Macquaria australasica. Conservation Genetics Resources. doi:10.1007/s12686-016-0666-0
Perez et al. 2017	No	Perez, C. R., Bonar, S. A., Amberg, J. J., Ladell, B., Rees, C., Stewart, W. T., … Cantrell, C. (2017). Comparison of American Fisheries Society Standard Fish Sampling Techniques and Environmental DNA (eDNA) for Characterizing Fish Communities in a Large Reservoir. North American Journal of Fisheries Management, (just-accepted). Retrieved from http://afs.tandfonline.com/doi/abs/10.1080/02755947.2017.1342721
Niemiller et al. 2017	No	Niemiller, M. L., Porter, M. L., Keany, J., Gilbert, H., Fong, D. W., Culver, D. C., … Taylor, S. J. (2017). Evaluation of eDNA for groundwater invertebrate detection and monitoring: a case study with endangered Stygobromus (Amphipoda: Crangonyctidae). Conservation Genetics Resources. doi:10.1007/s12686-017-0785-2
Muñoz-Colmenero et al. 201	No	Muñoz-Colmenero, M., Ardura, A., Clusa, L., Miralles, L., Gower, F., Zaiko, A., & Garcia-Vazquez, E. (2017). New specific molecular marker detects Ficopomatus enigmaticus from water eDNA before positive results of conventional sampling. Journal for Nature Conservation. doi:10.1016/j.jnc.2017.12.004

Mizumoto et al. 2017	No	Mizumoto, H., Urabe, H., Kanbe, T., Fukushima, M., & Araki, H. (2017). Establishing an environmental DNA method to detect and estimate the biomass of Sakhalin taimen, a critically endangered Asian salmonid. Limnology. doi:10.1007/s10201-017-0535-x
Minamoto et al. 2017	No	Minamoto, T., Uchii, K., Takahara, T., Kitayoshi, T., Tsuji, S., Yamanaka, H., & Doi, H. (2017). Nuclear internal transcribed spacer-1 as a sensitive genetic marker for environmental DNA studies in common carp Cyprinus carpio. Molecular Ecology Resources, 17(2), 324–333. doi:10.1111/1755-0998.12586
Minamoto et al. 2017	No	Minamoto, T., Fukuda, M., Katsuhara, K. R., Fujiwara, A., Hidaka, S., Yamamoto, S., … Masuda, R. (2017). Environmental DNA reflects spatial and temporal jellyfish distribution. PLOS ONE, 12(2), e0173073. doi:10.1371/journal.pone.0173073
Mauvisseau et al. 2017	No	Mauvisseau, Q., Coignet, A., Delaunay, C., Pinet, F., Bouchon, D., & Souty-Grosset, C. (2017). Environmental DNA as an efficient tool for detecting invasive crayfishes in freshwater ponds. Hydrobiologia. doi:10.1007/s10750-017-3288-y
Mauvisseau et al. 2017	No	Mauvisseau, Q., Parrondo, M., Fernández, M. P., García, L., Martínez, J. L., García-Vázquez, E., & Borrell, Y. J. (2017). On the way for detecting and quantifying elusive species in the sea: The Octopus vulgaris case study. Fisheries Research, 191, 41–48. doi:10.1016/j.fishres.2017.02.023
Lugg et al. 2017	No	Lugg, W. H., Griffiths, J., van Rooyen, A. R., Weeks, A. R., & Tingley, R. (2017). Optimal survey designs for environmental DNA sampling. Methods in Ecology and Evolution. doi:10.1111/2041-210X.12951
Larson et al. 2017	No	Larson, E. R., Renshaw, M. A., Gantz, C. A., Umek, J., Chandra, S., Lodge, D. M., & Egan, S. P. (2017). Environmental DNA (eDNA) detects the invasive crayfishes Orconectes rusticus and Pacifastacus leniusculus in large lakes of North America. Hydrobiologia, 800(1), 173–185. doi:10.1007/s10750-017-3210-7
Lance et al. 2017	No	Lance, R. F., Klymus, K. E., Richter, C. A., Guan, X., Farrington, H. L., Carr, M. R., … Baerwaldt, K. L. (2017). Experimental observations on the decay of environmental DNA from bighead and silver carps. Retrieved from http://www.reabic.net/journals/mbi/2017/ICAIS/MBI_2017_Lance_etal_correctedproof.pdf

Klobucar et al. 2017	No	Klobucar, S. L., Rodgers, T. W., & Budy, P. (2017). At the forefront: evidence of the applicability of using environmental DNA 1 to quantify the abundance of fish populations in natural lentic waters with additional sampling considerations. *Canadian Journal of Fisheries and Aquatic Sciences* .
Keller et al. 2017	No	Keller, S. R., Hilderbrand, R. H., Shank, M. K., & Potapova, M. (2017). Environmental DNA genetic monitoring of the nuisance freshwater diatom, Didymosphenia geminata , in eastern North American streams. Diversity and Distributions. doi:10.1111/ddi.12536
Katano et al. 2017	Yes	Katano, I., Harada, K., Doi, H., Souma, R., & Minamoto, T. (2017). Environmental DNA method for estimating salamander distribution in headwater streams, and a comparison of water sampling methods. PLOS ONE, 12(5), e0176541. doi:10.1371/journal.pone.0176541
Jo et al. 2017	No	Jo, T., Murakami, H., Masuda, R., Sakata, M. K., Yamamoto, S., & Minamoto, T. (2017). Rapid degradation of longer DNA fragments enables the improved estimation of distribution and biomass using environmental DNA. Molecular Ecology Resources. doi:10.1111/1755-0998.12685
Hutchins et al. 2017	No	Hutchins, P. R., Sepulveda, A. J., Martin, R. M., & Hopper, L. R. (2017). A probe-based quantitative PCR assay for detecting Tetracapsuloides bryosalmonae in fish tissue and environmental DNA water samples. Conservation Genetics Resources. doi:10.1007/s12686-017-0812-3
Hinlo et al. 2017	No	Hinlo, R., Gleeson, D., Lintermans, M., & Furlan, E. (2017). Methods to maximise recovery of environmental DNA from water samples. PLOS ONE, 12(6), e0179251. doi:10.1371/journal.pone.0179251
Hashizume et al. 2017	No	Hashizume, H., Sato, M., Sato, M. O., Ikeda, S., Yoonuan, T., Sanguankiat, S., … Minamoto, T. (2017). Application of environmental DNA analysis for the detection of Opisthorchis viverrini DNA in water samples. Acta Tropica, 169, 1–7. doi:10.1016/j.actatropica.2017.01.008
Guilfoyle et al. 2017	No	Guilfoyle, M. P., Farrington, H. L., Lance, R. F., Hanson-Dorr, K. C., Dorr, B. S., & Fischer, R. A. (2017). Movement of Hypophthalmichthys DNA in the Illinois River Watershed by the Double-Crested Cormorant (Phalacrocorax auritus). Waterbirds, 40(1), 63–68. doi:10.1675/063.040.0109

Gorički et al. 2017	No	Gorički, Š., Stanković, D., Snoj, A., Kuntner, M., Jeffery, W. R., Trontelj, P., … Aljančič, G. (2017). Environmental DNA in subterranean biology: range extension and taxonomic implications for Proteus. Scientific Reports, 7, 45054. doi:10.1038/srep45054
Gingera et al. 2017	No	Gingera, T., Bajno, R., Docker, M., & Reist, J. (2017). Environmental DNA as a detection tool for zebra mussels Dreissena polymorpha (Pallas, 1771) at the forefront of an invasion event in Lake Winnipeg, Manitoba, Canada. Management of Biological Invasions, 8(3), 287–300. doi:10.3391/mbi.2017.8.3.03
Gaynor et al. 2017	Yes	Gaynor, J. J., Bologna, P. A. X., Restaino, D. J., & Barry, C. L. (2017). qPCR Detection of Early Life History Stage Chrysaora quinquecirrha (Sea Nettles) in Barnegat Bay, New Jersey. Journal of Coastal Research, 78, 184–192. doi:10.2112/SI78-014.1
Gargan et al. 2017	No	Gargan, L. M., Morato, T., Pham, C. K., Finarelli, J. A., Carlsson, J. E. L., & Carlsson, J. (2017). Development of a sensitive detection method to survey pelagic biodiversity using eDNA and quantitative PCR: a case study of devil ray at seamounts. Marine Biology, 164(5). doi:10.1007/s00227-017-3141-x
Evans et al. 2017	No	Evans, N. T., Shirey, P. D., Wieringa, J. G., Mahon, A. R., & Lamberti, G. A. (2017). Comparative Cost and Effort of Fish Distribution Detection via Environmental DNA Analysis and Electrofishing. Fisheries, 42(2), 90–99. doi:10.1080/03632415.2017.1276329
Dysthe et al. 2017	Yes	Dysthe, J. C., Carim, K. J., Ruggles, M., McKelvey, K. S., Young, M. K., & Schwartz, M. K. (2017). Environmental DNA assays for the sister taxa sauger (Sander canadensis) and walleye (Sander vitreus). PLOS ONE, 12(4), e0176459. doi:10.1371/journal.pone.0176459
Dunn et al. 2017	No	Dunn, N., Priestley, V., Herraiz, A., Arnold, R., & Savolainen, V. (2017). Behavior and season affect crayfish detection and density inference using environmental DNA. Ecology and Evolution. doi:10.1002/ece3.3316
Doi et al. 2017	Yes	Doi, H., Uchii, K., Matsuhashi, S., Takahara, T., Yamanaka, H., & Minamoto, T. (2017). Isopropanol precipitation method for collecting fish environmental DNA: Isopropanol method for eDNA. Limnology and Oceanography: Methods. doi:10.1002/lom3.10161

Doi et al. 2017	Yes	Doi, H., Katano, I., Sakata, Y., Souma, R., Kosuge, T., Nagano, M., … Tojo, K. (2017). Detection of an endangered aquatic heteropteran using environmental DNA in a wetland ecosystem. Royal Society Open Science, 4(7), 170568. doi:10.1098/rsos.170568
Doi et al. 2017	Yes	Doi, H., Akamatsu, Y., Watanabe, Y., Goto, M., Inui, R., Katano, I., … Minamoto, T. (2017). Water sampling for environmental DNA surveys by using an unmanned aerial vehicle: Drone water sampling for eDNA. Limnology and Oceanography: Methods. doi:10.1002/lom3.10214
De Ventura et al. 2017	No	De Ventura, L., Kopp, K., Seppälä, K., & Jokela, J. (2017). Tracing the quagga mussel invasion along the Rhine river system using eDNA markers: early detection and surveillance of invasive zebra and quagga mussels. Management of Biological Invasions, 8(1), 101–112. doi:10.3391/mbi.2017.8.1.10
Clusa et al. 2017	No	Clusa, L., Miralles, L., Basanta, A., Escot, C., & García-Vázquez, E. (2017). eDNA for detection of five highly invasive molluscs. A case study in urban rivers from the Iberian Peninsula. *PloS One* , *12* (11), e0188126.
Carlsson et al. 2017	No	Carlsson, J. E. L., Egan, D., Collins, P. C., Farrell, E. D., Igoe, F., & Carlsson, J. (2017). A qPCR MGB probe based eDNA assay for European freshwater pearl mussel (Margaritifera margaritifera L.). Aquatic Conservation: Marine and Freshwater Ecosystems. doi:10.1002/aqc.2788
Carim et al. 2017	No	Carim, K. J., Dysthe, J. C., Young, M. K., McKelvey, K. S., & Schwartz, M. K. (2017). A Noninvasive Tool to Assess the Distribution of Pacific Lamprey (Entosphenus tridentatus) in the Columbia River Basin. PLOS ONE, 12(1), e0169334. doi:10.1371/journal.pone.0169334
Cai et al. 2017	No	Cai, W., Ma, Z., Yang, C., Wang, L., Wang, W., Zhao, G., … Yu, D. W. (2017). Using eDNA to detect the distribution and density of invasive crayfish in the Honghe-Hani rice terrace World Heritage site. PLOS ONE, 12(5), e0177724. doi:10.1371/journal.pone.0177724
Buxton et al. 2017	No	Buxton, A. S., Groombridge, J. J., Zakaria, N. B., & Griffiths, R. A. (2017). Seasonal variation in environmental DNA in relation to population size and environmental factors. Scientific Reports, 7, 46294. doi:10.1038/srep46294

Buxton et al. 2017	No	Buxton, A. S., Groombridge, J. J., & Griffiths, R. A. (2017). Is the detection of aquatic environmental DNA influenced by substrate type? *PloS One* , *12* (8), e0183371.
Brozio et al. 2017	No	Brozio, S., Manson, C., Gourevitch, E., Burns, T. J., Greener, M. S., Downie, J. R., & Hoskisson, P. A. (2017). Development and Application of an eDNA Method to Detect the Critically Endangered Trinidad Golden Tree Frog (Phytotriades auratus) in Bromeliad Phytotelmata. PLOS ONE, 12(2), e0170619. doi:10.1371/journal.pone.0170619
Bastos Gomes et al. 2017	No	Bastos Gomes, G., Hutson, K. S., Domingos, J. A., Chung, C., Hayward, S., Miller, T. L., & Jerry, D. R. (2017). Use of environmental DNA (eDNA) and water quality data to predict protozoan parasites outbreaks in fish farms. Aquaculture, 479, 467–473. doi:10.1016/j.aquaculture.2017.06.021
Baldigo et al. 2017	No	Baldigo, B. P., Sporn, L. A., George, S. D., & Ball, J. A. (2017). Efficacy of Environmental DNA to Detect and Quantify Brook Trout Populations in Headwater Streams of the Adirondack Mountains, New York. Transactions of the American Fisheries Society, 146(1), 99–111. doi:10.1080/00028487.2016.1243578
Balasingham et al. 2017	Yes	Balasingham, K. D., Walter, R. P., & Heath, D. D. (2017). Residual eDNA detection sensitivity assessed by quantitative real-time PCR in a river ecosystem. Molecular Ecology Resources, 17(3), 523–532. doi:10.1111/1755-0998.12598
Ardura et al. 2017	No	Ardura, A., Zaiko, A., Borrell, Y. J., Samuiloviene, A., & Garcia-Vazquez, E. (2017). Novel tools for early detection of a global aquatic invasive, the zebra mussel Dreissena polymorpha: Detection of Dreissena polymorpha from eDNA. Aquatic Conservation: Marine and Freshwater Ecosystems, 27(1), 165–176. doi:10.1002/aqc.2655
Andruszkiewicz et al. 2017	No	Andruszkiewicz, E. A., Sassoubre, L. M., & Boehm, A. B. (2017). Persistence of marine fish environmental DNA and the influence of sunlight. PLOS ONE, 12(9), e0185043. doi:10.1371/journal.pone.0185043
Agersnap et al. 2017	No	Agersnap, S., Larsen, W. B., Knudsen, S. W., Strand, D., Thomsen, P. F., Hesselsøe, M., … Møller, P. R. (2017). Monitoring of noble, signal and narrow-clawed crayfish using environmental DNA from freshwater samples. PLOS ONE, 12(6), e0179261. doi:10.1371/journal.pone.0179261

Annexe 2: Detection results of the eDNA traces from *Margaritifera margaritifera* in all mesocosm with all technical and natural replicates using the primers and probe targeting the COI.

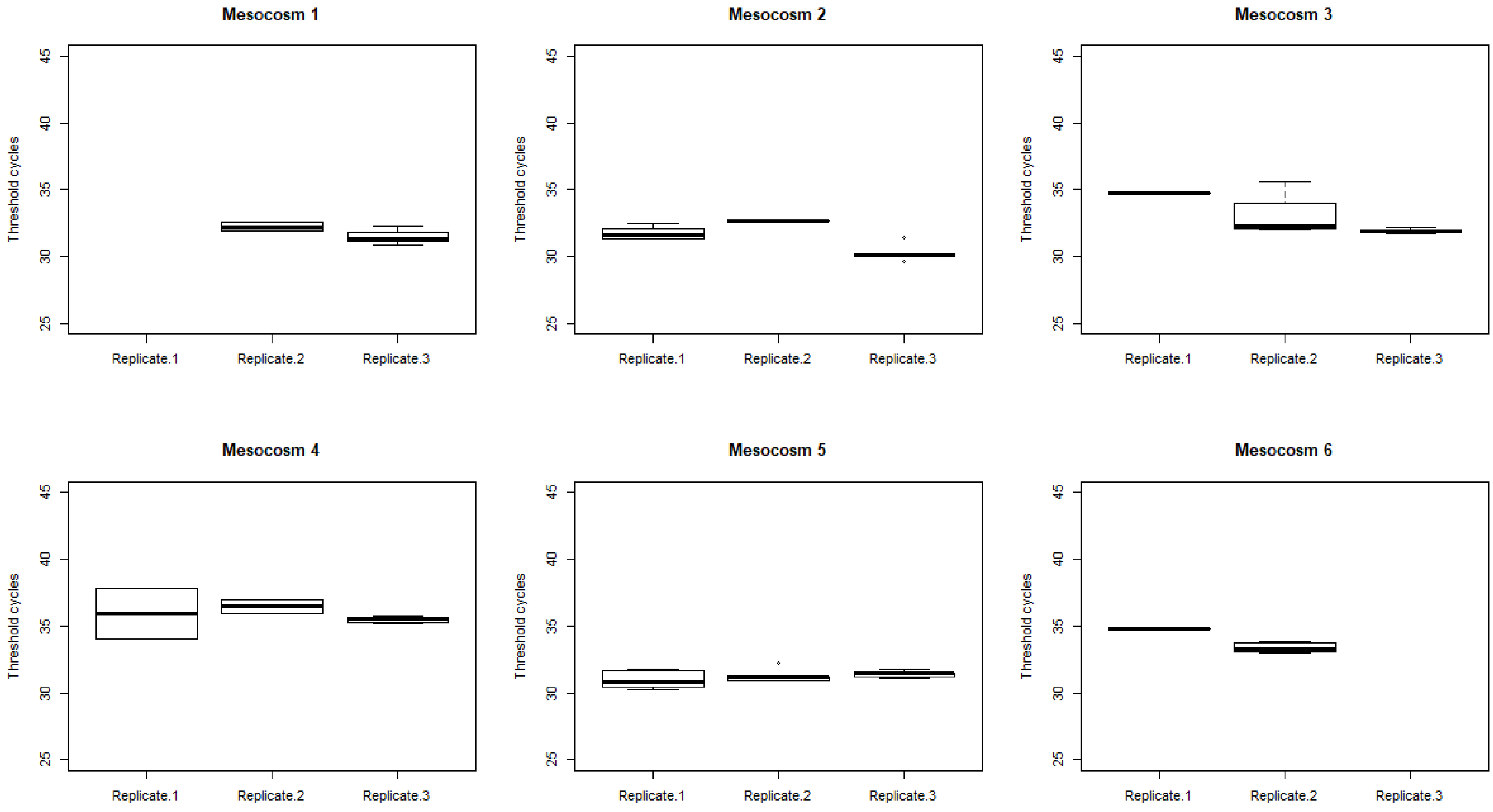

Annexe 3: Detection results of the eDNA traces from *Margaritifera margaritifera* in all mesocosm with all technical and natural replicates using the primers and probe targeting the 16S.

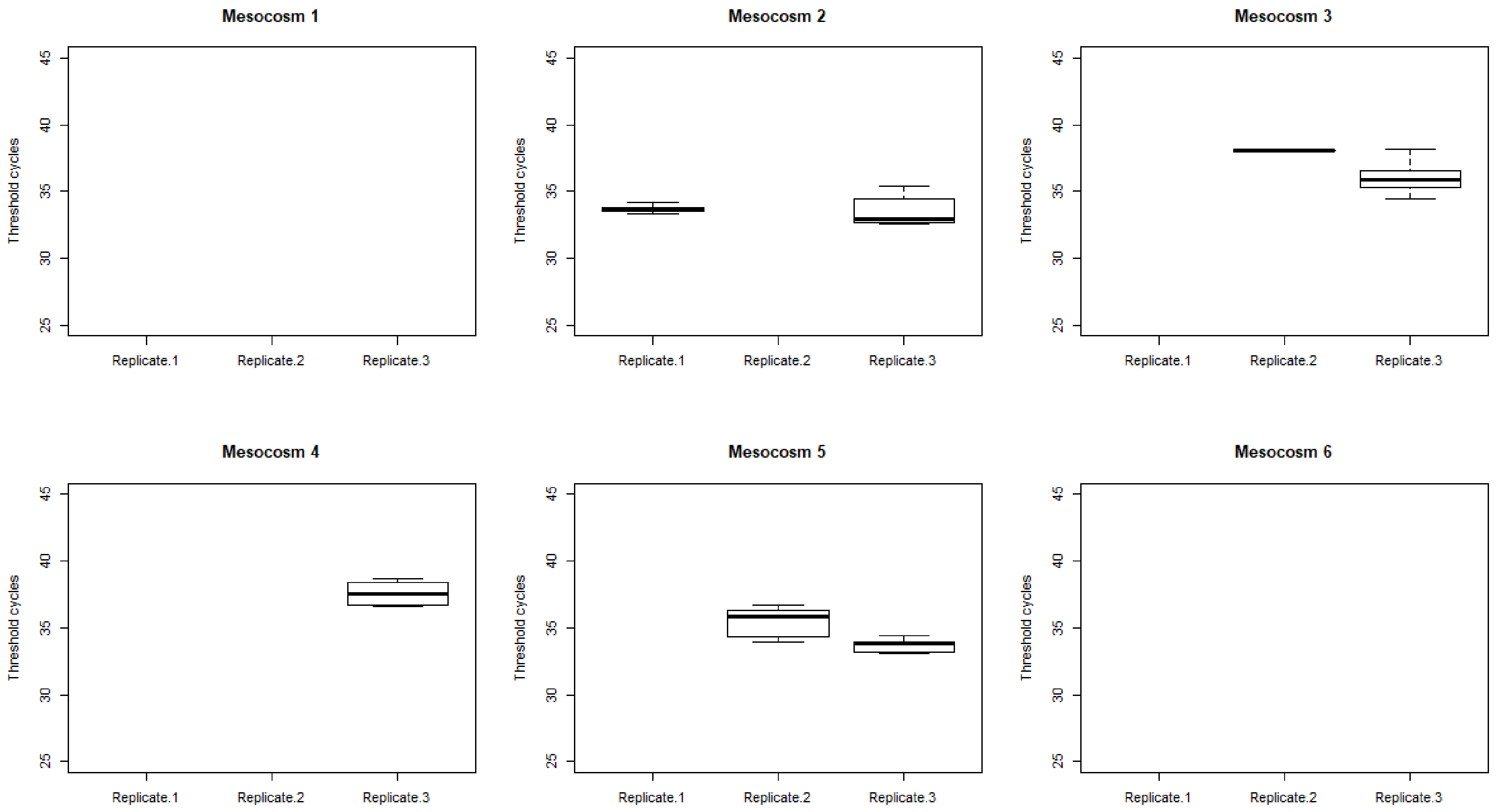

Annexe 4. MIQE Checklist

ITEM TO CHECK	IMPORTANCE	CHECKLIST	CHECKLIST
**EXPERIMENTAL DESIGN**		COI	16S
Definition of experimental and control groups	E	This study was performed in controlled mescosoms at the Freshwater Biological Association (FBA) station in Windermere	This study was performed in controlled mescosoms at the Freshwater Biological Association (FBA) station in Windermere
Number within each group	E	This study was performed in controlled mescosoms at the Freshwater Biological Association (FBA) station in Windermere	This study was performed in controlled mescosoms at the Freshwater Biological Association (FBA) station in Windermere
Assay carried out by core lab or investigator's lab?	D	Investigator's Lab	Investigator's Lab
Acknowledgement of authors' contributions	D	Yes	Yes
SAMPLE			
Description	E	Controlled mescosoms including breading population of the Freshwater Pearl Mussel *Margaritifera margaritifera*	Controlled mescosoms including breading population of the Freshwater Pearl Mussel *Margaritifera margaritifera*
Volume/mass of sample processed	D	Three 1L samples from each mesocosms	Three 1L samples from each mesocosms
Microdissection or macrodissection	E	N/A	N/A

Processing procedure	E	Three 1L water samples from each mesoscosms were took at the FBA for eDNA analysis in United Kingdom on 1st November 2017. Each water sample was filtered using a sterile 0.45 µm Sterivex™ HV filter (Sterivex™ filter unit, HV with luer-lock outlet, Merck®, Millipore®, Germany). The filters were stored at –80 °C until DNA extraction. DNA was extracted using Qiagen® DNA extraction Kit (DNeasy Blood & Tissue Kits) as per Spens et al. 2017.The final volume of an extracted sample was 150 µL with Buffer AE and stored at –20 °C until qPCR.	Three 1L water samples from each mesoscosms were took at the FBA for eDNA analysis in United Kingdom on 1st November 2017. Each water sample was filtered using a sterile 0.45 µm Sterivex™ HV filter (Sterivex™ filter unit, HV with luer-lock outlet, Merck®, Millipore®, Germany). The filters were stored at –80 °C until DNA extraction. DNA was extracted using Qiagen® DNA extraction Kit (DNeasy Blood & Tissue Kits) as per Spens et al. 2017.The final volume of an extracted sample was 150 µL with Buffer AE and stored at –20 °C [illegible]
If frozen - how and how quickly?	E	Filters were frozen at -20 immediately and then frozen at -80 back in the lab in Derby University	Filters were frozen at -20 immediately and then frozen at -80 back in the lab in Derby University
If fixed - with what, how quickly?	E	N/A	N/A
Sample storage conditions and duration (especially for FFPE samples)	E	All concentrated samples were stored in 1.5-mL microtubes at –20°C.	All concentrated samples were stored in 1.5-mL microtubes at –20°C.
NUCLEIC ACID EXTRACTION			
Procedure and/or instrumentation	E	We used DNA extraction kit from Qiagen DNeasy blood and tissue kit	We used DNA extraction kit from Qiagen DNeasy blood and tissue kit

Name of kit and details of any modifications	E	We used DNA extraction kit from Qiagen DNeasy blood and tissue kit as per Spens et al. 2017	We used DNA extraction kit from Qiagen DNeasy blood and tissue kit as per Spens et al. 2017
Source of additional reagents used	D	N/A	N/A
Details of DNase or RNAse treatment	E	N/A	N/A
Contamination assessment (DNA or RNA)	E	"Blank" filters with filtrated water from area when the targeted organism was not present and ddH2O was extracted with the eDNA samples from the mesocosm experiment. None of these blanks shows any amplification.	"Blank" filters with filtrated water from area when the targeted organism was not present and ddH2O was extracted with the eDNA samples from the mesocosm experiment. None of these blanks shows any amplification.
Nucleic acid quantification	E	Quantification for the standard curve was performed using a Nanodrop 2000 Spectrophotometer, (Thermofisher Scientific) following the manufacturer's instructions.	Quantification for the standard curve was performed using a Nanodrop 2000 Spectrophotometer, (Thermofisher Scientific) following the manufacturer's instructions.
Instrument and method	E		
Purity (A260/A280)	D		
Yield	D		
RNA integrity method/instrument	E	N/A	N/A
RIN/RQI or Cq of 3' and 5' transcripts	E	N/A	N/A
Electrophoresis traces	D	N/A	N/A
Inhibition testing (Cq dilutions, spike or other)	E	Not Checked	Not Checked
REVERSE TRANSCRIPTION			
Complete reaction conditions	E	N/A	N/A
Amount of RNA and reaction volume	E	N/A	N/A

Priming oligonucleotide (if using GSP) and concentration	E	N/A	N/A
Reverse transcriptase and concentration	E	N/A	N/A
Temperature and time	E	N/A	N/A
Manufacturer of reagents and catalogue numbers	D	N/A	N/A
Cqs with and without RT	D*	N/A	N/A
Storage conditions of cDNA	D	N/A	N/A
qPCR TARGET INFORMATION			
If multiplex, efficiency and LOD of each assay.	E	N/A	N/A
Sequence accession number	E	N/A	N/A
Location of amplicon	D	N/A	N/A
Amplicon length	E	83 bp	172bp
In silico specificity screen (BLAST, etc)	E	Primers and probes were found to be specific in silico using NCBI website (https://www.ncbi.nlm.nih.gov/) and Geneious Pro R10 software.	Primers and probes were found to be specific in silico using NCBI website (https://www.ncbi.nlm.nih.gov/) and Geneious Pro R10 software.
Pseudogenes, retropseudogenes or other homologs?	D	Not Found	Not Found
Sequence alignment	D	N/A	N/A
Secondary structure analysis of amplicon	D	Not Checked	Not Checked
Location of each primer by exon or intron (if applicable)	E	N/A	N/A
What splice variants are targeted?	E	N/A	N/A
qPCR OLIGONUCLEOTIDES			

Primer sequences	E	COI Forward 5'-TTGTTGATTCGTGCTGAGTTAGG -3' Carlsson et al. 2017<br>COI Reverse 5'-GCATGAGCCGTAACAATAACATTG- 3' Carlsson et al. 2017	16S Forward 5'-CAACCCTGGAACCGCTAAAG -3' Stoeckle et al. 2015<br>16S Reverse 5'-GGCTGCGCTCATGTGAATTA -3' Stoeckle et al. 2015
RTPrimerDB Identification Number	D	Not Submitted	Not Submitted
Probe sequences	D**	COI Probe 6-FAM-CCTGGTTCTTTGCTGGGT -BHQ-1 Carlsson et al. 2017	16S Probe 6-FAM-TCCAGTTAATCATAGAACTTCATCAAA-BHQ-1 This study
Location and identity of any modifications	E		
Manufacturer of oligonucleotides	D	(Sigma-Aldricht) Merck KGaA, Darmstadt, Germany	(Sigma-Aldricht) Merck KGaA, Darmstadt, Germany
Purification method	D	HPLC	HPLC
qPCR PROTOCOL			
Complete reaction conditions	E	Reactions were set up manually in a specific cabinet using designated equipment.	Reactions were set up manually in a specific cabinet using designated equipment.
Reaction volume and amount of cDNA/DNA	E	Reaction volume is 25 µL, and amount of DNA is 3 µL	Reaction volume is 25 µL, and amount of DNA is 3 µL
Primer, (probe), Mg++ and dNTP concentrations	E	qPCR amplification was performed in a final volume of 25 µl using 15 µl of PrecisionPlus qPCR Master Mix with ROX (Primer Design, UK), 1 µl of each primer (10 µM), 1 µl of the corresponding probe (2.5 µM), 6.5 µl of ddH2O and 3µl of extracted DNA	qPCR amplification was performed in a final volume of 25 µl using 15 µl of PrecisionPlus qPCR Master Mix with ROX (Primer Design, UK), 1 µl of each primer (10 µM), 1 µl of the corresponding probe (2.5 µM), 6.5 µl of ddH2O and 3µl of extracted DNA

Polymerase identity and concentration	E	We used PrecisionPlus qPCR Master Mix with ROX (Primer Design, UK)	We used PrecisionPlus qPCR Master Mix with ROX (Primer Design, UK)
Buffer/kit identity and manufacturer	E	We used PrecisionPlus qPCR Master Mix with ROX (Primer Design, UK)	We used PrecisionPlus qPCR Master Mix with ROX (Primer Design, UK)
Exact chemical constitution of the buffer	D	N/A	N/A
Additives (SYBR Green I, DMSO, etc.)	E	N/A	N/A
Manufacturer of plates/tubes and catalog number	D	MicroAmp® Fast 96-Well Reaction plate (0,1ml) Applied Biosystems, Warrington, UK with optical adhesive covers Applied Biosystems, Warrington, UK	MicroAmp® Fast 96-Well Reaction plate (0,1ml) Applied Biosystems, Warrington, UK with optical adhesive covers Applied Biosystems, Warrington, UK
Complete thermocycling parameters	E	qPCR targeting the COI was warm up at 50°C for 2 min and denaturation at 95°C for 10 min, followed by 55 cycles 95°C for 15s, 60°C for 1 min	qPCR targeting the 16S included an initial denaturation at 95°C for 15s, followed by 55 cycles of 95°C for 15s, 60°C for 10s and 72°C for 20s
Reaction setup (manual/robotic)	D	We performed following the manufacturer's instructions.	We performed following the manufacturer's instructions.
Manufacturer of qPCR instrument	E	ABI StepOnePlus™ Real-Time PCR (Applied Biosystems, Warrington, UK).	ABI StepOnePlus™ Real-Time PCR (Applied Biosystems, Warrington, UK).
qPCR VALIDATION			
Evidence of optimisation (from gradients)	D	Not Performed	Not Performed

Specificity (gel, sequence, melt, or digest)	E	The specificity of the primers-probes targeting the COI and 16S were tested against DNA extracted from Margaritifera margaritifera (Linnaeus 1758), Margaritifera falcata (Gould, 1850), Anodonta anatina (Linnaeus, 1758), Anodonta cygnea (Linnaeus, 1758), Unio pictorum (Linnaeus, 1758), Dreissena rostriformis bugensis (Andrusov, 1897), Dreissena polymorpha (Pallas, 1771), Corbicula fluminea (Müller, 1774), Truncilla truncata (Rafinesque, 1820), Quadrula quadrula (Rafinesque, 1820), Lampsilis siliquoidea (Barnes, 1823) and Cumberlandia monodonta (Say, 1829). [illegible]	The specificity of the primers-probes targeting the COI and 16S were tested against DNA extracted from Margaritifera margaritifera (Linnaeus 1758), Margaritifera falcata (Gould, 1850), Anodonta anatina (Linnaeus, 1758), Anodonta cygnea (Linnaeus, 1758), Unio pictorum (Linnaeus, 1758), Dreissena rostriformis bugensis (Andrusov, 1897), Dreissena polymorpha (Pallas, 1771), Corbicula fluminea (Müller, 1774), Truncilla truncata (Rafinesque, 1820), Quadrula quadrula (Rafinesque, 1820), Lampsilis siliquoidea (Barnes, 1823) and [illegible]
For SYBR Green I, Cq of the NTC	E	N/A	
Standard curves with slope and y-intercept	E	Slope: (-3,042) , y-intercept: 28,238	Slope: -3,767 , y-intercept: 30,413
PCR efficiency calculated from slope	E	113,14%	84,28%
Confidence interval for PCR efficiency or standard error	D	Not Checked	
r2 of standard curve	E	0.9852 for the COI assay	0.9739 for the 16S assay
Linear dynamic range	E	Not Checked	
Cq variation at lower limit	E	The positive signals were detected from at least one of the six qPCR replicates per eDNA sample	The positive signals were detected from at least one of the six qPCR replicates per eDNA sample

Confidence intervals throughout range	D	Not Checked	Not Checked
Evidence for limit of detection	E	Based on the standard	Based on the standard
If multiplex, efficiency and LOD of each assay.	E	N/A	N/A
DATA ANALYSIS			
qPCR analysis program (source, version)	E	StepOnePlus™ Software v2.2.2	StepOnePlus™ Software v2.2.2
Cq method determination	E	We performed according to default setting of Software above.	We performed according to default setting of Software above.
Outlier identification and disposition	E		
Results of NTCs	E	At least four wells of no-template negative control were adopted in all qPCR plates for both assays.	At least four wells of no-template negative control were adopted in all qPCR plates for both assays.
Justification of number and choice of reference genes	E	N/A	N/A
Description of normalisation method	E	We used standard curve methods.	We used standard curve methods.
Number and concordance of biological replicates	D	N/A	N/A
Number and stage (RT or qPCR) of technical replicates	E	We performed in 6 replicate for each eDNA samples. We performed the standard curve with 10 replicates for each dilution.	We performed in 6 replicate for each eDNA samples. We performed the standard curve with 10 replicates for each dilution.
Repeatability (intra-assay variation)	E	Not Checked	Not Checked
Reproducibility (inter-assay variation, %CV)	D	Not Checked	Not Checked
Power analysis	D	N/A	N/A
Statistical methods for result significance	E	We performed according to default setting of Software above.	We performed according to default setting of Software above.
Software (source, version)	E	StepOnePlus™ Software v2.2.2	StepOnePlus™ Software v2.2.2

Cq or raw data submission using RDML	D	Not Submitted	Not Submitted
